# The soil‐borne legacy in the age of the holobiont

**DOI:** 10.1111/1751-7915.13325

**Published:** 2018-10-16

**Authors:** Linda S. Thomashow, Melissa K. LeTourneau, Youn‐Sig Kwak, David M. Weller

**Affiliations:** ^1^ Wheat Health, Genetics, and Quality Research Unit United States Department of Agriculture Agricultural Research Service Pullman WA 99164‐6430 USA; ^2^ Department of Plant Medicine and Institute of Agriculture & Life Sciences Gyeongsang National University Jinju 52828 Korea

## Abstract

Future efforts to increase agricultural productivity will focus on crops as functional units comprised of plants and their associated microflora in the context of the various environments in which they are grown. It is suggested that future efforts to increase agricultural productivity will focus on crops as functional units comprised of plants and their associated beneficial microorganisms in the context in which they are grown. Scientists, industry, and farmers must work closely together to develop, adapt, and apply new technologies to a wide range of cropping systems. Consumer education is needed help grow public awareness that ‘plant probiotics’ offer a safe and environmentally friendly alternative to dependence on the use of chemical pesticides.

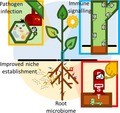

The need for increased agricultural productivity to meet the food, fibre, feed and fuel requirements of the growing population is indisputable, as is the challenge of attaining this goal with minimal impact on diminishing land and water resources. At first glance, the burden of greater productivity will fall largely on the shoulders of farmers and the agricultural industry as a whole, but producers worldwide already are adopting state‐of‐the‐art management practices that are part of precision agriculture, including the use of information technology and automated equipment to lessen the waste of seed, fertilizer, fuel, and the overuse of pesticides. Also gaining recognition among farmers and their industrial partners is that a much better understanding of soil quality is central to agricultural productivity. This will lead to wider soil testing and increasingly precise delivery of water and other inputs tailored to localized needs on a plot‐by‐plot basis. Still wanting, however, is recognition that soil quality encompasses not only a soil's chemical‐physical properties, but also its rich microbiological component, a soil‐borne legacy that acts to shape plant growth, enhance plant nutrient uptake, improve root architecture, and provide a first line of defence against biotic and abiotic stresses manifested under sometimes harsh conditions.

The plant‐associated microbiota and in particular, the microorganisms residing near, on and within the root are as integral to plant health as the gut microbiota are to our own well‐being. Plants and their associated microbes have evolved together over 400 million years, but we have only recently begun to appreciate how profoundly their association can be exploited to maximize agricultural productivity, conserve natural resources and reduce the need for chemical inputs. The future will see tremendous progress towards revealing the rich legacy of these soil‐borne microbes, and three overlapping lines at the forefront of current investigation are especially likely to generate fundamental new knowledge that can be applied to agricultural systems worldwide. These areas of research focus on the induction of specific soil suppressiveness to soil‐borne pathogens, the induction of systemic resistance by ‘immunized’ or ‘primed’ plants and the identification of beneficial microbiota indigenous to the plant endosphere. Specific soil suppressiveness is a long‐known natural mechanism of disease control that typically occurs after several years of monoculture of certain crops and involves selection by the plants of beneficial microbiota after a severe outbreak of a soil‐borne disease. Particular bacterial and fungal species have been implicated in some examples of specific soil suppressiveness, but evidence is accumulating that newly described endosphere communities also have a key role in these special situations. Knowing the identity of the beneficial rhizobacteria and/or endophytes and the mechanisms involved in their selection by plants will enable the development of crop inoculants that will permit growers to eliminate the disease phase in generating suppressive soils. Similarly, induced systemic resistance in plants, first recognized in 1991, results from the presence of particular rhizobacteria, a soil‐borne legacy that primes the plant immune system for enhanced defence responses when the plant is subsequently challenged by a broad range of pathogens or insect herbivores. Knowing the molecular signature of the sensitizing stimulus and the signals that strengthen and maintain the longevity of the associated rhizobacteria could provide long‐term foliar disease resistance and growth promotion to stimulated crops. The third line of intensive study, the role of endophytes in growth promotion and plant protection, will continue to show how these novel soil‐borne microbes ultimately take up residence and benefit the plant host from within its tissues. The insights to be gained from these and other ongoing studies, and the tools to be developed in the coming decade, will reveal intricate physiological and biochemical networks of interaction among microbes, and between them and their hosts, at the most intimate level. These advances will enable environmental microbiologists to participate directly in the revolution in agricultural productivity that is already underway and must continue to accelerate towards fulfilling the needs of future populations.

## Towards a functionally defined rhizobiome

The past decade has seen exponential growth in the use of genomic sequencing to access the bacterial richness and diversity within the rhizobiome, herein considered to include the root and the microbial taxa in habitats adjacent to, on or inside it. Microbial community composition within these habitats differs according to the identity of the host plant, the proximity to its interior and its developmental stage over a range of cultural conditions and environmental stresses. Genomic sequence analyses have led to recognition both of the overarching role of the environment in shaping the rhizobiome and the presence of core taxa that are maintained consistently over progressively greater temporal, spatial, environmental and evolutionary scales and therefore are considered to reflect significant interdependence between the plant and its microbial partners. However, the characterization of microbial communities based on sequence analyses of marker genes like 16S rRNA provides only a snapshot of the relative abundance of the organisms detected and is constrained by inadequate reference databases, untested functional inferences and the paucity of data with which to capture interactions among plant‐associated taxa. The coming years will see these gaps addressed by metagenomic and single cell sequencing technologies that will lead to improved databases, enhancing the quality of inferred community structural and functional interactions among taxa. More efficient methods for the recovery of expressed RNA from environmentally complex samples will permit routine transcriptomic and metatranscriptomic analyses of dynamic interactions among taxa in their native habitats. Greater emphasis will be placed on the absolute rather than the relative abundance of rhizobiota, and physiological and biochemical relationships predicted *in silico* will be substantiated *in vitro* and *in planta* by complementary culture‐based studies. Improved growth media and novel devices that enable the isolation *in situ* of microbial species not readily recovered *in vitro* will be applied to access bacteria that until now have evaded cultivation. Many such microbes are not recovered by conventional techniques but can be ‘domesticated’ in culture, which will open the way to understanding their contribution to complex processes like plant nutrient acquisition and defence. Collectively, the application of functionally oriented studies of the rhizobiome will yield insight not only into what organisms are present, but also will bridge the gap towards understanding their role in plant growth, development, physiology and stress tolerance.

## It takes a community

**Figure 1:**
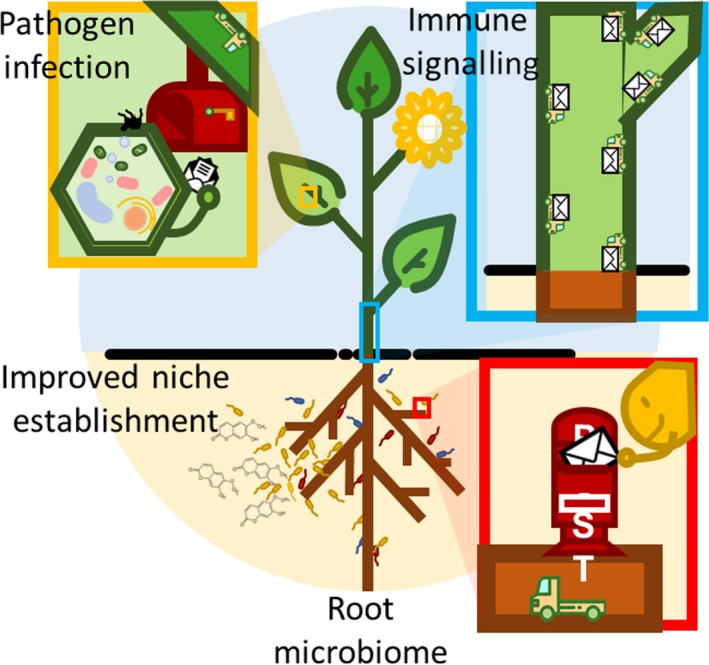
A primed leaf cell (upper left) under attack by bacterial pathogen (black cells) sends a cry for help to the roots, initiating defence signalling (envelopes). Roots in iron‐poor soil excrete scopoletin, enhancing niche establishment for *P. simiae* WCS417 (yellow cells). Illustration: G. J. Cho.

A functional holobiont, defined here as the plant and its associated microbiota, requires communication mediated by molecular signals that carry out cross‐talk among the resident microbes and with the host plant. Insight into the identity of the chemical messages involved will be gained by metabolomic analyses not only of the nutrients and signals present in root extracts or exudates, but also of those produced by microbes stimulated by the presence of each other, the root, and the soil environment. These studies will, when coupled with genomics and transcriptomics, provide a holistic understanding of the interactions between the plant and the complex microbial communities it nurtures. Metabolic fingerprinting already has shown how foliar stimulation of host plant systemic disease resistance also activates the host iron deficiency response in the roots, leading to greater exudation of an iron‐scavenging phenolic compound that helps to provide both improved niche establishment for microbial partners on the roots and growth and immunity benefits for the plant (Fig. 1; Stringlis *et al*., 2018). Such studies will be extended to identify key signals present in the environmentally relevant concentrations exchanged between plants and other microbes, beneficial or deleterious, that have known effects on plant health. Future investigations also will focus on signals within and between more complex rhizobacterial assemblages selected by the plant itself and capable of stimulating disease suppression and growth promotion even in subsequent plant generations (Schlatter *et al*., 2017; Berendsen *et al*., 2018). Additional insights will be gained by dissecting communication within the synthetic communities (SynComs) predicted by complex neural networks, and that give rise to beneficial plant attributes (Herrera Paredes *et al*., 2018). Insights will be gained into how compounds like antimicrobials, volatiles, quorum‐sensing signals, and interactions based on resource preference, competition or nutritional interdependence shape relationships even in communities that modulate the biosynthesis of plant‐derived natural products active against bacterial and fungal plant pathogens, nematodes and herbivores. Microbial colonization also can induce changes in the plant morphology and its metabolome, qualitatively and quantitatively altering the synthesis of phytohormones, flavours, fragrances and compounds of potential pharmaceutical value (Etalo *et al*., 2018). Technological advances from empirical and modelled research also will enable the extension of analyses to more natural conditions and with agronomically relevant host plants influenced by the chemical complexity of native soils, yielding a foundation of principles that can be applied to the diverse and changing environments under which agriculture is practiced. In the long term, such information will be essential to the development of biofertility and biocontrol inoculants, giving rise to consistently functioning microbial communities that enhance crop productivity, as well as in the breeding of crop varieties better able to support a healthy rhizobiome.

## Multi‐scale interactions

Greater consistency in the performance of introduced microorganisms in the future will necessitate a more holistic perspective that addresses not only the beneficial agents and the metabolites they produce, but also the micro‐scale biogeographical parameters that modulate their interactions *in situ*. Efforts to understand rhizosphere processes that are amplified over time and space to produce dramatic long‐term field‐scale effects are currently confounded by reliance upon bulk methods alone. Future studies will make greater use of model systems and powerful analytical tools that provide insight into microbial interactions on the scale at which they occur in nature. Such model systems range from simple agar surfaces that reveal how location in a colony, aggregate or biofilm impacts metabolism and diffusion to influence microbial interactions, to micro‐3D printed platforms responsive to the distances and cell numbers that enable effective signalling within and between bacterial aggregates. Modern confocal laser scanning microscopes and highly refined analytical approaches also will be used to visualize micro‐scale interactions. Many of these new methodologies combine stable isotope probing (SIP) with DNA‐ or RNA‐based *in situ* hybridization, Raman microspectroscopy or high resolution secondary ion mass spectrometry (nano‐SIMS) and *in situ* ion beam‐scattering electron microscopy to generate insights into nutrient cycling within biofilms and on root surfaces. These and other approaches will be applied to address the impact of rhizosphere bioactivity with minerals and on carbon storage and stabilization in soil organic matter (SOM), an essential component of soil health and fertility in agroecosystems. Microbial biomass and released metabolites comprise a significant fraction of SOM, and microbially mediated reactions in soil aggregates will receive increased attention in relation to field‐scale crop management practices directed towards more sustainable agricultural intensification and profitability. Electron microscopy has been a dedicated tool for imaging the interactions between microbe‐mineral interfaces in geomicrobiology, and new advances in focused ion beam milling (FIB) coupled with scanning and cryoelectron microscopy and complementary redox‐sensitive and synchrotron‐based methods such as X‐ray absorption near‐edge spectroscopy (XANES), scanning transmission X‐ray microscopy (STXM) and micro‐X‐ray fluorescence (micro‐XRF) will yield a clearer understanding of how microbially driven redox reactions mediate the availability of elemental plant nutrients such as N, P and Fe in soil aggregates and at the interface between the rhizosphere and the bulk soil.

## Concluding thoughts

We envision that future efforts to increase agricultural productivity will focus on crops as holobionts, functional units comprised of plants and their associated microflora in the context of the various environments in which they are grown. Success in this endeavour will inevitably necessitate changes in how agriculture in general, and the soil‐borne legacy in particular, are perceived at levels ranging from the lab to the field. Microbiologists and geneticists, plant physiologists, soil scientists and bioinformaticians will work in concert to develop novel concepts; industry will transform concepts into innovative deliverables; and farmers will accept and apply the resultant biotechnological products and advances in infrastructure and information technology over the full range of cropping systems. At the farm level, more organic and ecofriendly management strategies, the evolving paradigm of soil health, and persisting soil and water conservation concerns will draw attention to opportunities to enhance food production and quality in agroecosystems managed to support rhizosphere function. At the same time, consumer acceptance of beneficial rhizobacteria as ‘plant probiotics’, and recognition of the added value of sustainably produced commodities, will benefit local agricultural communities through greater profitability of farming systems and changes that support cultural institutions and social values.
